# Supplementary material for: Rockfish: A transformer-based model for accurate 5-methylcytosine prediction from nanopore sequencing
Source: Nat Commun. 2024 Jul 3;15:5580. doi: 10.1038/s41467-024-49847-0 (PMC11222435; doi:10.1038/s41467-024-49847-0)
Supplement: Supplementary file 1 — Supplementary Information [file 41467_2024_49847_MOESM1_ESM.pdf]

# Supplementary material

## Running Guppy (R9.4.1)

Guppy is a basecalled developed by ONT. It is used for canonical base calling by methylation calling tools. The guppy version used in all experiments (for R9.4.1. data) is 5.0.14, with a super-accurate model. An example of the command is:

```
guppy_basecaller -i <fast5_folder> -r -s <output_folder> \
    --config dna_r9.4.1_450bps_sup.cfg --device <devices> \
    --fast5_out # Used for storing move table
```

## Running Dorado (R10.4.1)

For R10.4.1 data we used a new basecalling tool developed by ONT. The version used for basecalling was 0.4.0. DNA model used for canonical basecalling (and Remora modification calling) is dna_r10.4.1_e8.2_400bps_sup@v4.2.0 with Remora 5mCG_5hmCG compatible modification model.

```
dorado basecaller \
    -x <devices> -r --emit-moves \ # Emit moves for Rockfish
    --reference <reference_fasta> \
    --secondary=no -N 0 -Y \
    --modified-bases 5mCG_5hmCG --modified-bases-threshold 0.0 \ # Only for Remora
    <dna_r10.4.1_e8.2_400bps_sup@v4.2.0_model_path> \
    <pod5s> > basecalls.bam
```

## Running Megalodon

Megalodon is a methylation-calling tool developed by ONT. If Rerio model is used, canonical and modification basecalling is done jointly. When utilizing the Remora backend, canonical basecalling is performed using Guppy and modification basecalling is done by Remora. Megalodon improves Rerio's (or Remora's) performance by anchoring basecalling output to a reference. First, a basecalled sequence is aligned onto the reference genome. Next, every candidate (5mC in CpG on a read-level) modification is evaluated against the canonical base using Viterbi scoring. The position is labelled as modified if the score corresponding to the modified base is higher than the score corresponding to the canonical base. Otherwise, the candidate is labelled as unmodified. Megalodon version used for all experiments is 2.4.2, with Rerio (res_dna_r941_min_modbases_5mC_CpG_v001) or Guppy (5.0.14) and Remora (0.1.2) used as backends. Minimap (v2.24) is provided via mappy. An example of a Megalodon command is:

```
megalodon \
    <fast5_folder> \
    --guppy-config dna_r9.4.1_450bps_sup.cfg \
    --guppy-server-path <guppy_basecall_server_path> \
    --remora-modified-bases dna_r9.4.1_e8 sup 0.0.0 5mc CG 0 \
    --outputs per_read_mods mods \
```

```
--write-mods-text \
--reference <reference_fasta> \
--devices <gpus> \ # format: cuda:d_0,d_1,… (e.g. cuda:0,1,2)
--processes <n_processes>
```

## Running Nanopolish

Nanopolish is a methylation-calling tool based on the hidden Markov model (HMM). First, basecalled reads (fastq) are aligned using minimap2 (v2.24). Next, the raw nanopore signal corresponding to the aligned read is aligned to the reference sequence (event_align). For every candidate group (a region around one or multiple CpGs) Nanopolish calculates the ratio between the likelihood of methylation and the likelihood of unmethylation. If the ratio > 0, the region is labelled as methylated, otherwise as unmethylated.
Nanopolish version used for all experiments is 0.14.0. Commands used for running Nanopolish are given here:

1. Nanopolish index:

```
nanopolish index -d <fast5_folder> <reads>
```

2. Nanopolish align:

```
minimap2 -t <threads> -a -x map-ont <reference> <reads> | samtools sort -T tmp -o <bam>
samtools view -@ <threads> -F 2308 -bS <in_bam> > <out_bam>
samtools index -@ <threads> <bam>
```

3. Nanopolish call methylation:

```
nanopolish call-methylation -r <reads> -b <bam> \
  -g <reference> -q cpg -s reference \
  -t <threads> --min-mapping-quality 0 > <tsv_output>
```

Site-level frequency was calculated using the script available at:
https://github.com/jts/nanopolish/blob/v0.14.0/scripts/calculate_methylation_frequency.py
Since Nanopolish calls methylation for a region (not for individual position), all read-level calls are extracted using the algorithm that can be found in the code above (lines 52-67).

## Running Modkit (extraction of Remora's methylation calls)

Link: https://github.com/nanoporetech/modkit
Version: v0.2.4

### Read-level results:

```
modkit extract -t <THREADS> --mapped-only --kmer-size 1 --reference <REFERENCE> \
 --cpg --no-filtering -p 0 --ignore h <IN_BAM> <OUT_PATH>
```

### Site-level results:

```
modkit pileup -t <THREADS> --ignore h --cpg -r <REFERENCE_FASTA> --only-tabs \
 <IN_BAM> <OUT_BED>
```

## Rockfish installation and inference

Rockfish can be installed by cloning the GitHub repository (#TODO Add repo) and invoking the pip install command:

```
git clone ... rockfish && cd rockfish
pip install --extra-index-url https://download.pytorch.org/whl/cu113 .
```

The user should replace "cu113" (in the link) with the desired CUDA version (e.g. for cuda 10.2, the link is https://download.pytorch.org/whl/cu102).

Rockfish model can be downloaded using the command:

```
rockfish download -m <{all, base, small}> -s <save_path>
```

The argument "`all`" will download both the base and small models.

An example of Rockfish inference:

```
rockfish inference -i <basecalled/workspace> --reference <reference> \
  --model_path <model_path> -r -t <n_workers> \
  -b <batch_size> \  # Total batch size (all GPUs), default 8092
  -d <devices>  # Format: d_0,d_1,... (e.g.) "0,1,2"
```

Note: The input to the inference is a workspace folder that can be found in the folder saved by Guppy. A workspace folder contains fast5 files annotated with fastq and move table.

## Rockfish training dataset generation

To train new models, users can follow these steps:

1. Data basecalling (Guppy or Dorado) – see above
2. Generate bedGraph (WGBS pipeline) – see below
3. Feature extraction (and optional merging):

```
#R9.4.1
rockfish extract -r -t <n_threads> --delete_src \
<path_to_pod5s> <reference_path> <output_path>


#R10.4.1
rockfish extract --bam_path <path_to_bam> -r -t <n_threads> \
--delete_src <path_to_pod5s> <output_path>
```

4. Generate ground-truth labels (for training and validation separately):

```
#R9.4.1
python scripts/extract_training_examples.py --rf <data_file> \
  --bedgraph <bedgraph_file> --exclude_ctgs chrM --balanced --seed 42


#R10.4.1
python scripts/extract_ref_pos.py --minq 2 --workers <n_processes> \
```

```
  <bam> <reference> > mappings.tsv
python scripts/extract_training_examples.py --rf <data_file> \
  --mappings <mappings_file>  --bedgraph <bedgraph_file> \
  --exclude_ctgs chrM --balanced --seed 42 \
  --output_rf <rf_output_file> --output_labels <labels_file>
```

5. Data merging (optional, if there are multiple files):

```
rockfish merge [input_rf_files ...] <dest_file>
cat [label_files ....] > <merged_labels>
```

6. Run the training:

```
# Index rockfish file (for training, validation...)
rockfish index <rf_file>

PYTHONPATH=<path_to_rockfish_folder> python -m rockfish.model.model fit \
  --data.train_data <train_rf_file> --data.train_labels <train_labels> \
  --data.val_data <val_rf_file> --data.val_labels <val_labels> \
  --trainer.strategy=DDPStrategy \
  --trainer.strategy.find_unused_parameters=False \
  --trainer.accelerator gpu --trainer.precision 16-mixed \
  --trainer.gradient_clip_val 1.0 --model.n_layers 12 \
  --model.features 256 --model.dim_ff 2048 \
  --data.train_batch_size 1024 --data.val_batch_size 2048 \
  --trainer.val_check_interval 10_000 --model.separate_unk_mask False \
  --model.bases_mask_prob 0.2 --model.wd 0.001 --model.alpha 0.05 \
  --model.block_size 6 --model.signal_mask_prob 0 \
  --seed_everything=42 --trainer.max_steps 2_000_000
```

Note: For "rockfish" commands, run "--help" to see the full list of command-line arguments and their explanations.

## WGBS pipeline

The pipeline for processing bisulfite data is similar to the pipeline given by ONT (https://ont-open-data.s3.amazonaws.com/gm24385_mod_2021.09/bisulphite/fastq2bed.sh). The pipeline consists of five steps:

1. Adapter and quality trimming using trim_galore
2. Alignment using bismark
3. Deduplication using deduplicate_bismark
4. Methylation extraction using bismark_methylation_extractor
5. Converting output from 4) to bedGraph using bismark2bedGraph

In the first step we use "--three_prime_clip_R1 15 --clip_R2 15" arguments only for the GM24385 dataset. Moreover, deduplication (third step) was skipped for NA19240 since it is recommended to skip deduplication for RRBS (https://github.com/FelixKrueger/Bismark/issues/234).

## Ablation study

The ablation study was done using an R9.4.1 Rockfish base model with 4 encoder and 4 decoder layers due to limited resources. Ablated models were trained on the whole training dataset (as described in Methods) and evaluated on NA24835 Chromosome 1. The results demonstrated the importance of the alignment decoder in both read- and site-level prediction. The accuracy dropped by 0.02 and 0.0003 for read- and site-level respectively after removing the decoder. The auxiliary loss of predicting masked bases, as well as masking the input bases without the associated base masking loss were consistently proven as important on the read- and site-level. The differences between the accuracies of the base and the ablated models were 0.0163 and 0.0021 on the read-level, and 0.0002 and 0.0001 on the site-level. Finally, the results obtained after removing the auxiliary tasks related to signal masking, as well as the additional signal encodings were comparable to the base model at the read- and site-level. There was no noticeable drop in the accuracy of the models. Although a slight improvement of 0.0002 on the read-level was noticed when removing the additional encodings, that did not transfer to the site-level accuracy.

Similarly, the correlation between Rockfish models and WGBS is reduced after ablating the model. In line with previous results, the biggest difference comes from removing the decoder, while the removal of the additional encodings, and auxiliary tasks related to signal masking shows only a slight decline in correlation results (Supplementary Figure S7). Since all ablated components that did not exhibit noticeable significance except for the additional encodings are not utilized in the reference but rather only during the training, an end-user would not experience any effects of removing those components. The differences between the ablated models and the full model could become more obvious with model upscaling (e.g. when using 12 encoder and 12 decoder layers).

## Resource utilization

Resource utilization analysis was performed by comparing the running time, CPU, memory and GPU usage of all ONT-based tools. The K562 dataset was utilized to compare R9.4.1 ONT-based models. Megalodon Remora and Megalodon Rerio call methylation in an end-to-end fashion (basecalling, alignment and methylation calling), so only one command is run. Nanopolish pipeline requires running four sequential commands: basecalling, indexing, alignment and methylation calling. Rockfish currently requires running two consecutive commands: basecalling and methylation calling. The mean running time for all commands used for every R9.4.1 ONT-based tool is given in Supplementary Figure 9 and in Supplementary Table 5. As expected, due to its lower model complexity, Megalodon Rerio is the fastest tool (mean running time of 3143 s) followed by Megalodon Remora (12475 s) with a running time similar to Guppy canonical basecalling (11751 s). The Rockfish base model is the slowest (25331 s). The Rockfish small model (14727 s) is slightly faster than Nanopolish (14953 s) and reduces methylation calling time by 78.09% compared with the base model. In the pipeline, which includes the small model, Guppy was the bottleneck taking 79.79% of the total running time. The batch sizes used for the evaluation were 8192 for the base model and 16384 for Rockfish small.

To compare running time of R10.4.1 Rockfish model with Remora, a subset of 400 000 reads was sampled from the R10.4.1 NA12878 dataset. Furthermore, two instances of Rockfish were compared, one with a traditional attention implementation, and the other with a technically advanced flash attention (FA). Remora requires running a single command that calls methylation in an end-to-end manner while Rockfish, same as in the R9.4.1 case, requires running basecalling and methylation calling in a consecutive manner. The mean running times of all commands used by R10.4.1 ONT-based tools are gives in Supplementary Figure 10 and Supplementary Table 11. Remora (7028 s) is faster than both versions of Rockfish models (17 774 s Rockfish without FA, 12 059 Rockfish with FA).

Furthermore, we evaluated the resource utilization of all R9.4.1 (Supplementary Table 5) and R10.4.1 ONT-based tools (Supplementary Table 11). All tools utilize less than 32 CPU-s on average in each phase. The peak memory usage estimates are the lower bound in the multiprocessing use-case since it is obtained by tracking the memory usage of the main process and all child processes the main process was waiting for. All R9.4.1 ONT-based tools achieve similar peak memory usage: Megalodon Remora (36421 MB), Megalodon Rerio (31859 MB), Nanopolish (27136 MB), Rockfish base (29347 MB) and Rockfish small (31071 MB). Megalodon Remora and Rockfish models (including Guppy's basecalling step) have very high mean GPU utilization (>90%) while Megalodon Rerio's mean GPU utilization is ~68%. Finally, Rockfish models have the highest peak GPU memory utilization (peak GPU memory usage is 68% for Rockfish base and 72% for Rockfish small) compared with Megalodon Remora (54%) and Megalodon Rerio (43%). Nanopolish, being a CPU tool, was not included in the GPU analysis. R10.4.1 Remora achieves peak memory usage of 38992 MB. Rockfish models achieve peak memory usage of 45479 MB during Dorado basecalling while the methylation calling step achieves peak of 4265 MB for Rockfish without FA and 8790 for Rockfish with FA. R10.4.1 models achieve very high mean GPU-utilization (all steps have mean GPU-utilization of >99% except for methylation calling of Rockfish with FA which achieves mean of ~98%). Finally, R10.4.1 Rockfish variants have higher peak GPU memory usage of 69% while compared with Remora’s 44%.

# Supplementary Figures

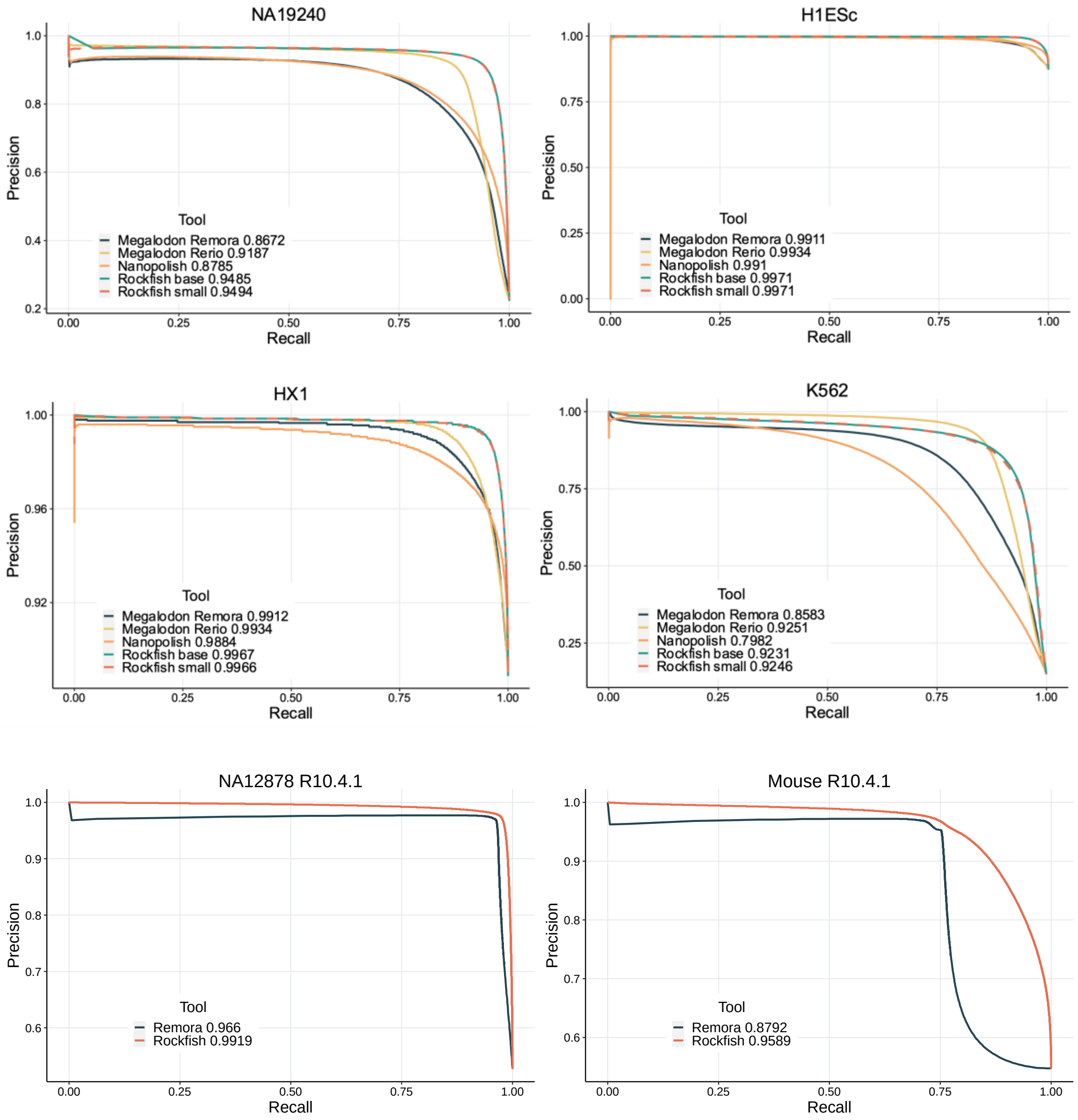


*Figure S1: Precision-recall curves on the read-level evaluation for **a)** NA19240, **b)** H1Esc, **c)** HX1, **d)** K562 **e)** NA12878 R10.4.1 and **f)** neonatal mouse R10.4.1 datasets. Only examples predicted by every ONT tool are included. Included predictions are intersected with WGBS data. Partially methylated positions and positions with coverage less than x5 (WGBS) are excluded. Rockfish and Megalodon examples are sorted using modification probabilities. Nanopolish is sorted using the log-likelihood ratio value. Source data are provided as a Source Data file.*

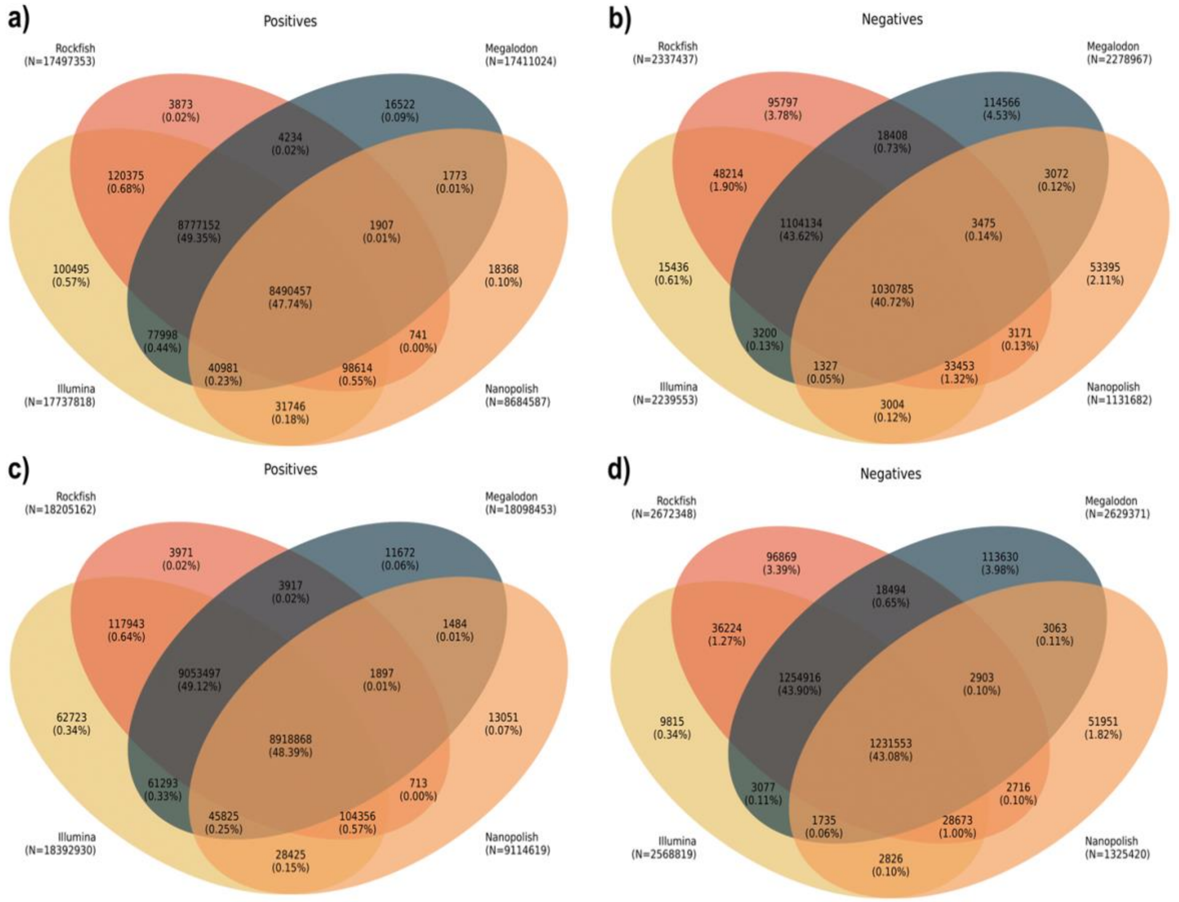


*Figure S2: Venn diagrams representing predicted positives and negatives for HX1 (**a-b**) and H1ESc (**c-d**) datasets. Actual positives and negatives (the ground truth) are given by the set named "Illumina". Rockfish is represented with the small model. Sample space is defined as the set of all fully unmethylated or methylated sites called by Illumina with at least 5x. Rockfish calls the highest amount of true positives and true negatives and achieves high precision and recall. Rockfish also calls the least false positives and less false negatives compared to Megalodon.*

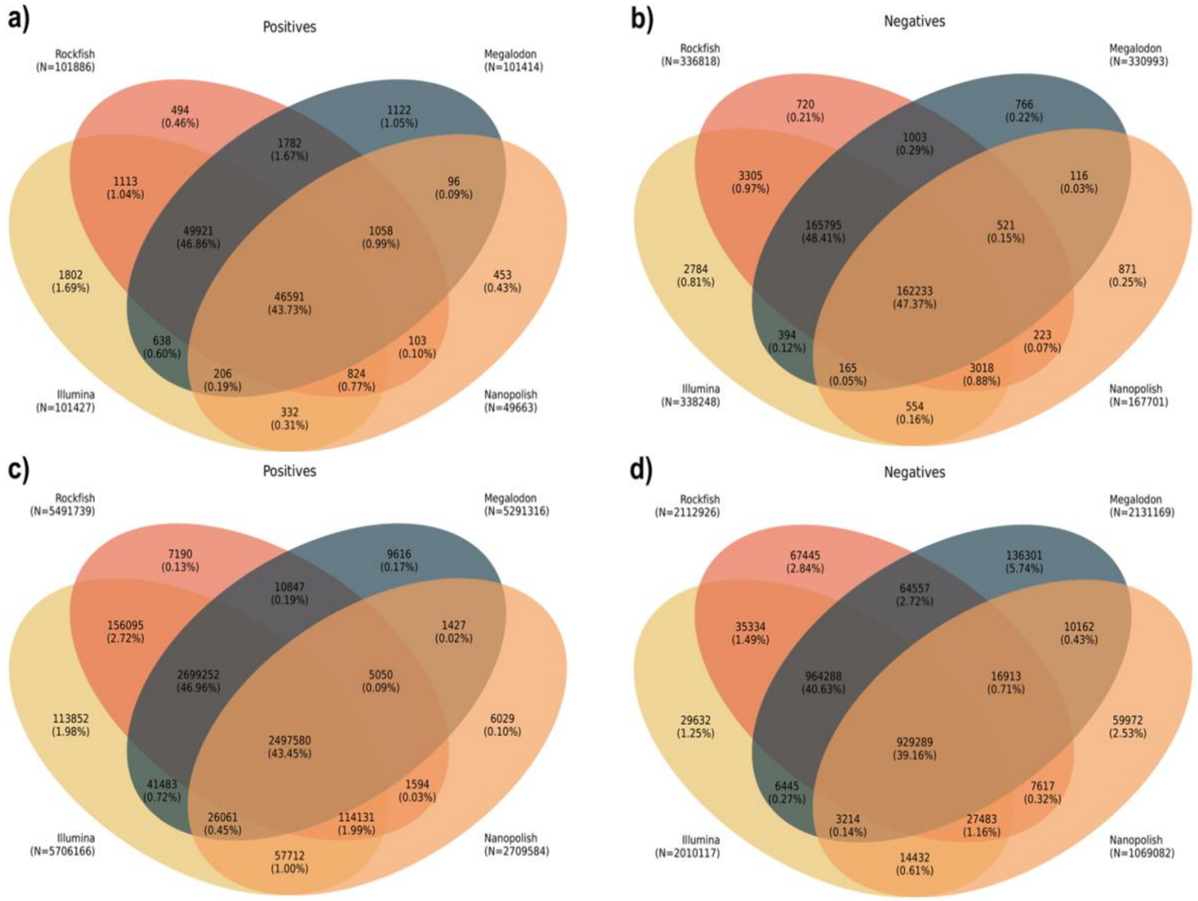


*Figure S3: Venn diagrams representing predicted positives and negatives for NA19240 (**a-b**) and R9.4.1 C57BL/6 Neonatal mouse (**c-d**) datasets. Actual positives and negatives (the ground truth) are given by the set named "Illumina". Rockfish is represented with the small model. Sample space is defined as the set of all fully unmethylated or methylated sites called by Illumina with at least 5x. Rockfish calls significantly more true positives and negatives. Rockfish calls fewer false positives compared to Megalodon, but slightly more less negatives than Megalodon for NA19240.*

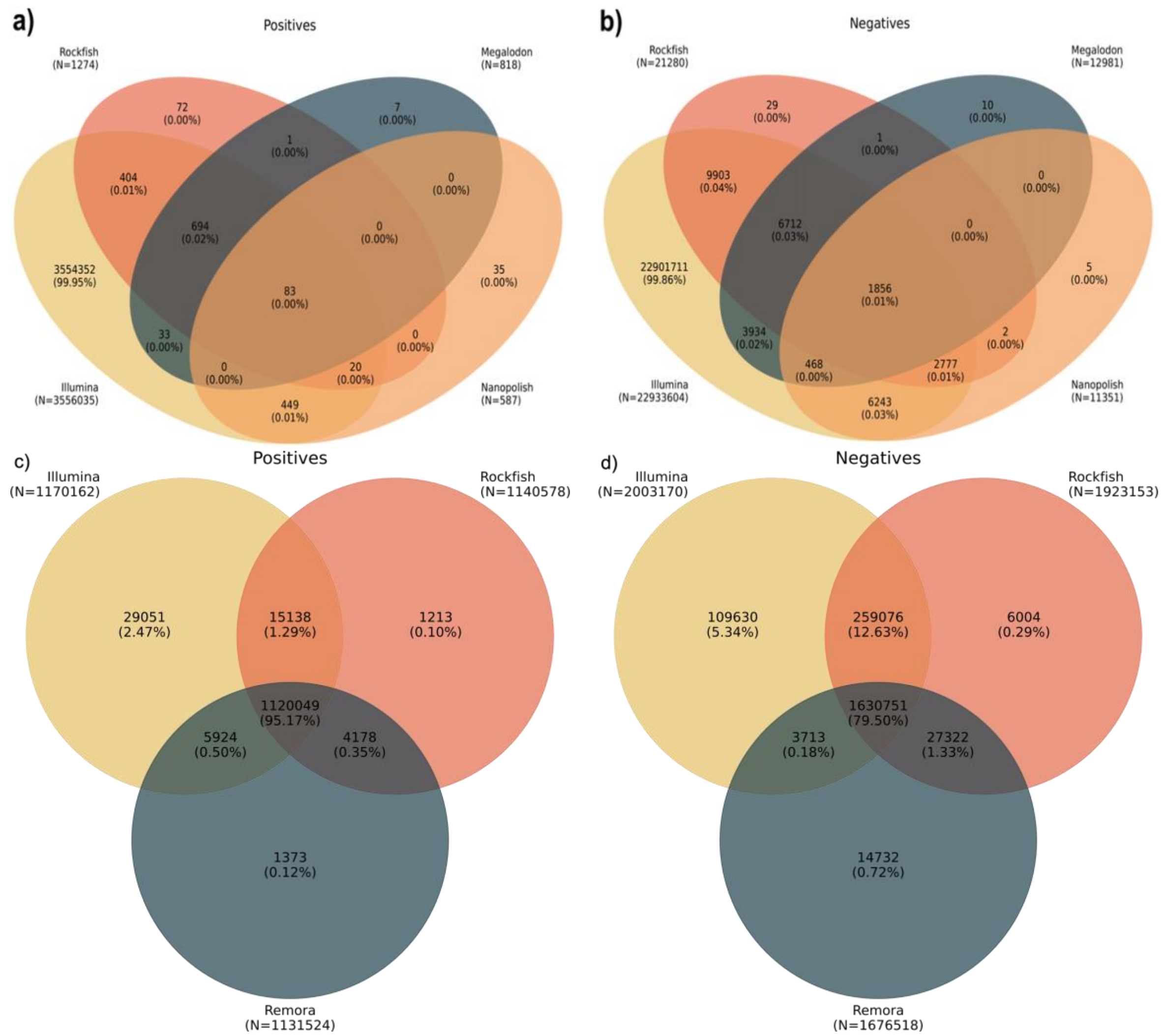


*Figure S4: Venn diagrams representing predicted positives and negatives for the K562 (**a-b**) and Neonatal mouse R10.4.1 (**c-d**) datasets. Actual positives and negatives (the ground truth) are given by the set named "Illumina". For K562 dataset (R9.4.1) Rockfish is represented with the small model. Sample space is defined as the set of all fully unmethylated or methylated sites called by Illumina with at least 5x. For K562, an overwhelming majority of examples are not called by the ONT methods due to low ONT coverage.*

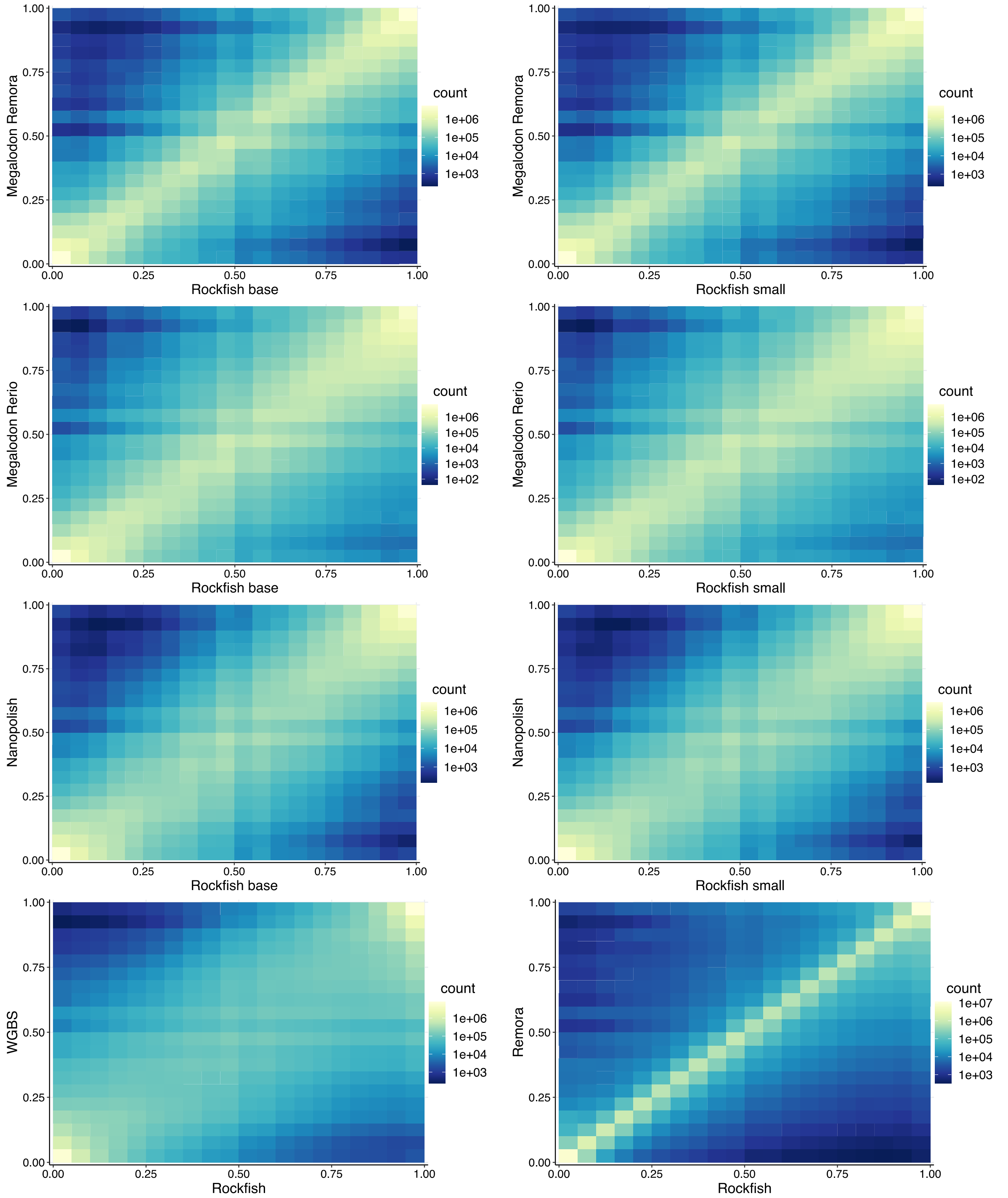


*Figure S5: 2D histograms showing Pearson's correlation between Rockfish models and other ONT-based tools for the NA12878 R9.4.1 dataset (**a-f**) and for the NA12878 R10.4.1 dataset (**g-h**). Each axis is divided into 20 bins, with counts being plotted on a log scale. Subfigure a) shows the correlation between Rockfish base model and Megalodon Remora (Pearson's r = 0.9573), b) Rockfish small and Megalodon Remora (r = 0.9584), c) Rockfish base model and Megalodon Rerio (r = 0.9309), d) Rockfish small and Megalodon Rerio (r = 0.9317), e) Rockfish base and Nanopolish (r = 0.9190), f) Rockfish small and Nanopolish (r = 0.9202), g) Rockfish and WGBS (r = 0.9093) and h) Rockfish and Remora (r = 0.9785). P-value = 0 for all tests. Source data are provided as a Source Data file.*

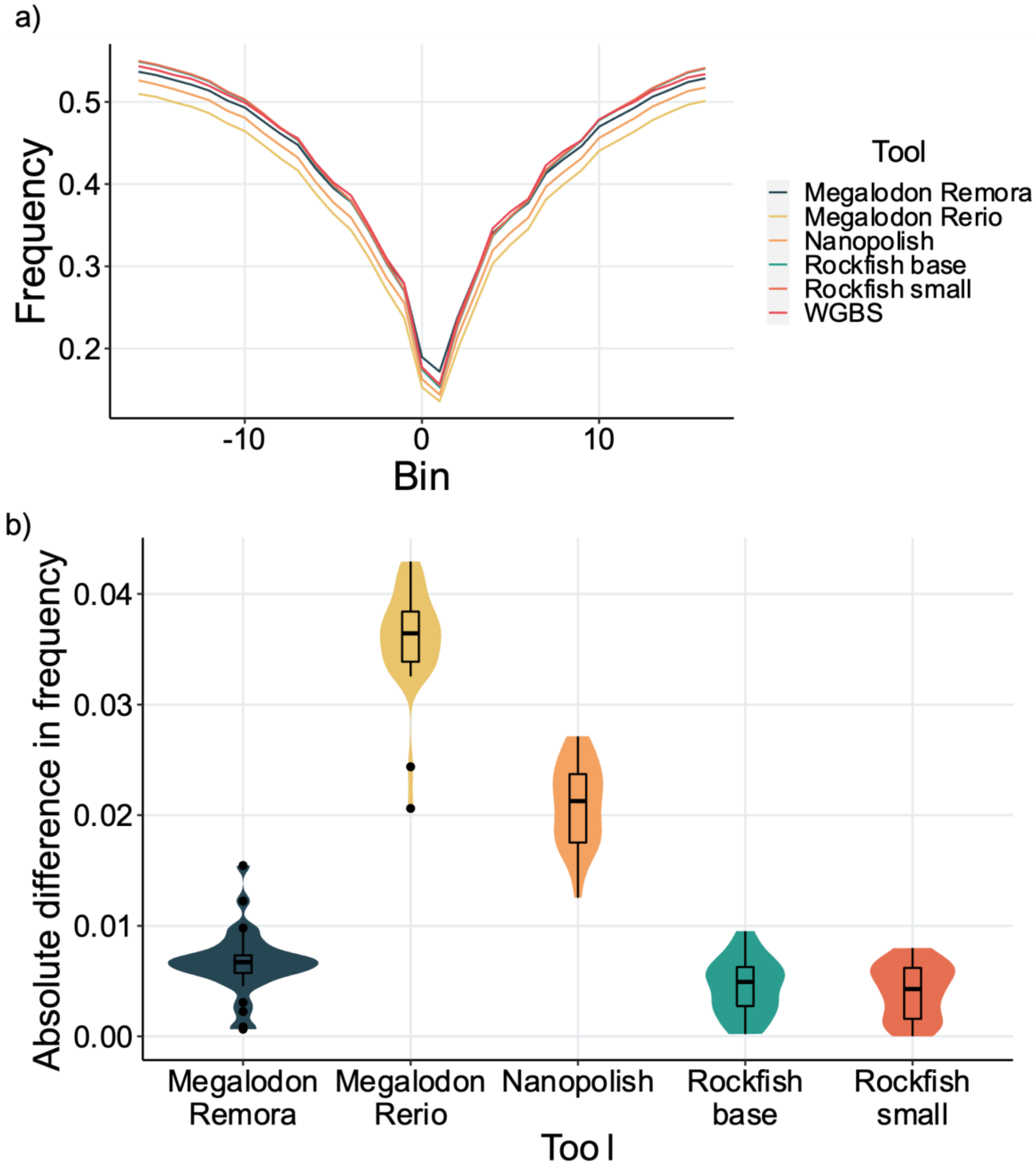


*Figure S6: a) Methylation frequency for every ONT-based tool and WGBS with respect to the binned distance from the start of CTCF binding peaks on the NA12878 R9.4.1 dataset. Both Rockfish models show high consistency with other ONT-based tools and, more importantly, WGBS. b) The distribution of the absolute difference for every ONT-based tool and WGBS. Rockfish models reduce the absolute difference between ONT and WGBS. Data (n=33) in the box plot are presented as follows: the centre line indicates the median, the bounds of the box represent the first and third quartiles (Q1 and Q3), and the whiskers extend to the minimum and maximum values within 1.5 times the interquartile range (IQR). Outliers beyond this range are plotted individually. Source data are provided as a Source Data file.*

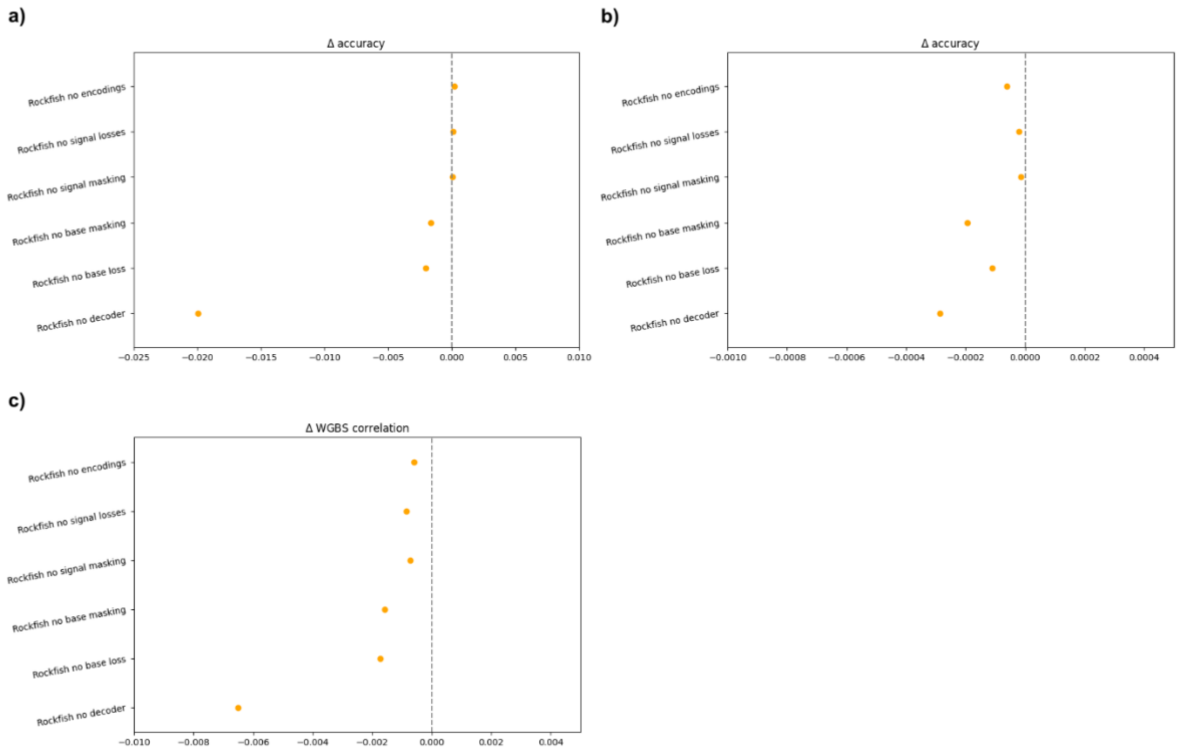


*Figure S7: Ablation analyses showing the significance of different Rockfish components. The accuracies of the ablated models are compared to the accuracy of the full model, and the absolute values for a) read-level prediction, b) site-level prediction, and c) site-level correlation with WGBS are plotted. The full model is not shown since it corresponds to 0 Δ accuracy and 0 Δ WGBS correlation. The decoder proves to be the most important component in all three analyses. On the other hand, auxiliary signal masking-based tasks seem not to have a significant role in accurate methylation calling which might be due to performing the ablation using a model with reduced dimensionality (4 vs 12 encoder and decoder layers). Source data are provided as a Source Data file.*

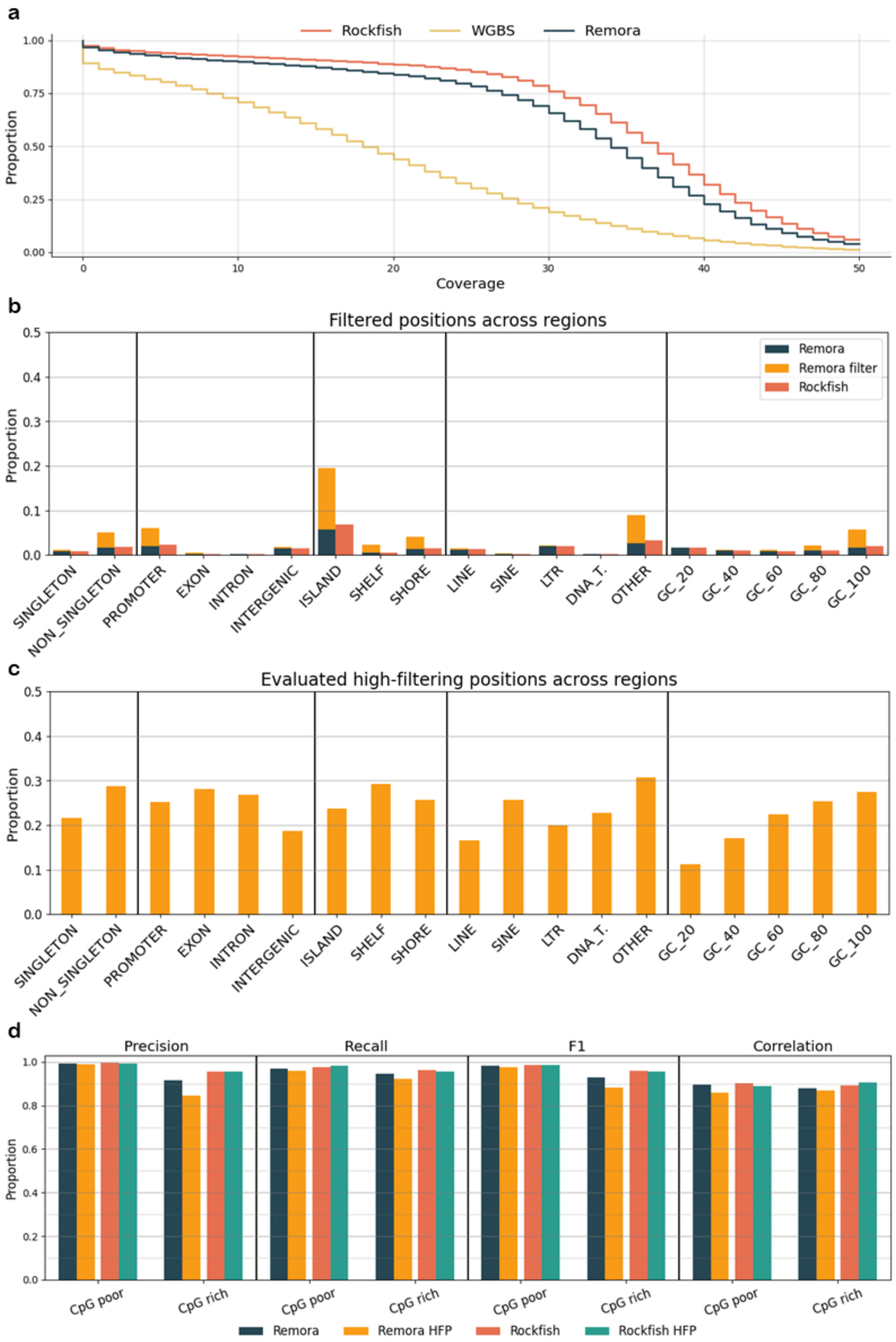


*Figure S8: The analysis of Remora's filtering effects in the R10.4.1 Mouse dataset. a) Complementary cumulative distribution function (CCDF) of the strand-specific calling coverage for each ONT-based method and WGBS for the R10.4.1 Mouse dataset shows that Rockfish achieves noticeably higher mean coverage compared with Remora (Rockfish 22.65x vs Remora 20.69x). b) Distribution of proportions of positions not evaluated due to low coverage with the distinction between positions with overall coverage below threshold and positions with overall coverage above threshold but valid coverage below in Remora's case. c) Distribution of proportions of high-filtering positions (HFP) with sufficient valid coverage but above the expected*

*number of filtered calls >10%. d) Site-level evaluation in CpG poor and CpG rich promoter regions for all positions and high-filtering positions (HFP). Source data are provided as a Source Data file.*

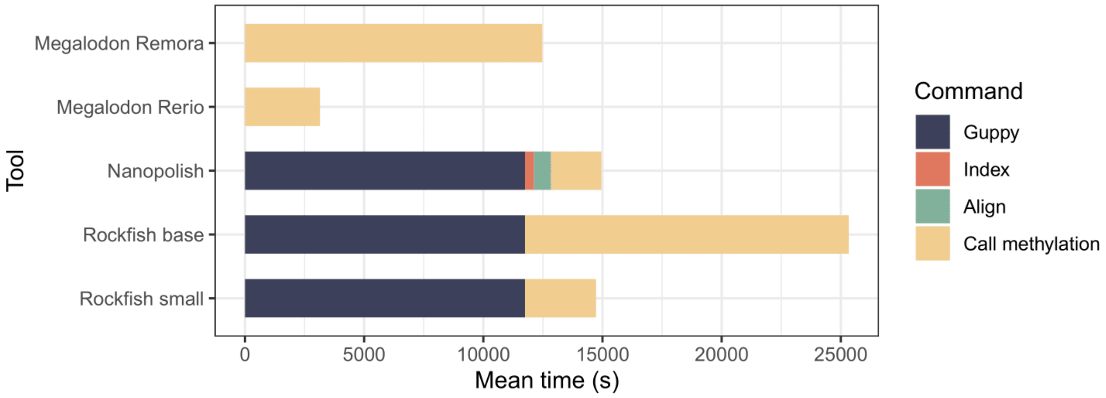


*Figure S9: Mean running time for each R9.4.1 ONT-based tool on the K562 dataset. Megalodon models require invoking only one command which includes basecalling, alignment and methylation calling. Rockfish requires invoking two commands - basecalling and inference. The inference consists of both alignment and methylation calling. Nanopolish requires invoking four commands. Different command types are plotted in different colours. Each command is called three times. Source data are provided as a Source Data file.*

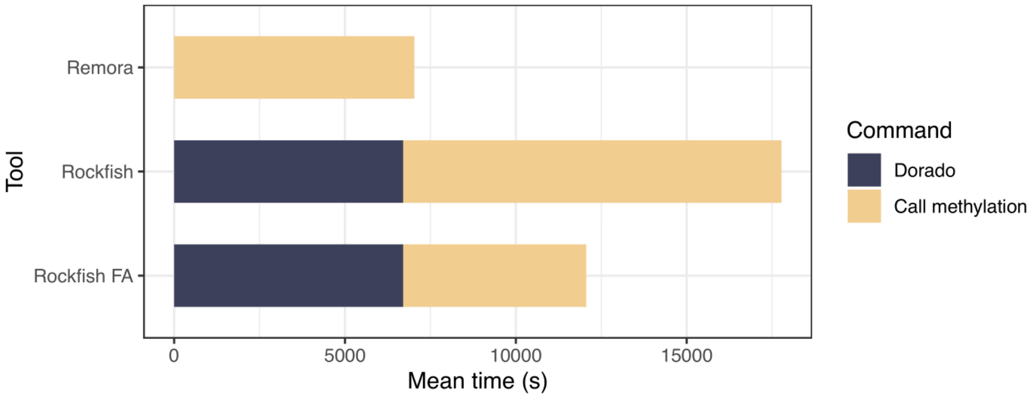


*Figure S10: Mean running time for R10.4.1 ONT-based tools on the subset of NA12878 R10.4.1 data. 100 pod5 files were used. Remora requires invoking only one command which includes basecalling, alignment and methylation calling. Rockfish requires invoking two commands - basecalling and inference. Two Rockfish instances correspond to the model with a traditional attention implementation (denoted as Rockfish) and the model including a technical advanced fast attention (denoted as Rockfish FA). The inference consists of both alignment and methylation calling. Basecalling and inference commands are plotted in different colours. Each command is called three times. Source data are provided as a Source Data file.*
