# Supplementary material for: Rockfish: A transformer-based model for accurate 5-methylcytosine prediction from nanopore sequencing
Source: Nat Commun. 2024 Jul 3;15:5580. doi: 10.1038/s41467-024-49847-0 (PMC11222435; doi:10.1038/s41467-024-49847-0)
Supplement: Supplementary file 2 — Reporting Summary [file 41467_2024_49847_MOESM2_ESM.pdf]

nature portfolio

Corresponding author(s): Mile Šikić

Last updated by author(s): Jun 5, 2024

Reporting Summary

Nature Portfolio wishes to improve the reproducibility of the work that we publish. This form provides structure for consistency and transparency in reporting. For further information on Nature Portfolio policies, see our Editorial Policies and the Editorial Policy Checklist.

Statistics

For all statistical analyses, confirm that the following items are present in the figure legend, table legend, main text, or Methods section.

n/a	Confirmed	
[ ]	[x]	The exact sample size (*n*) for each experimental group/condition, given as a discrete number and unit of measurement
[x]	[ ]	A statement on whether measurements were taken from distinct samples or whether the same sample was measured repeatedly
[ ]	[x]	The statistical test(s) used AND whether they are one- or two-sided<br>*Only common tests should be described solely by name; describe more complex techniques in the Methods section.*
[x]	[ ]	A description of all covariates tested
[x]	[ ]	A description of any assumptions or corrections, such as tests of normality and adjustment for multiple comparisons
[ ]	[x]	A full description of the statistical parameters including central tendency (e.g. means) or other basic estimates (e.g. regression coefficient) AND variation (e.g. standard deviation) or associated estimates of uncertainty (e.g. confidence intervals)
[ ]	[x]	For null hypothesis testing, the test statistic (e.g. *F*, *t*, *r*) with confidence intervals, effect sizes, degrees of freedom and *P* value noted<br>*Give P values as exact values whenever suitable.*
[x]	[ ]	For Bayesian analysis, information on the choice of priors and Markov chain Monte Carlo settings
[x]	[ ]	For hierarchical and complex designs, identification of the appropriate level for tests and full reporting of outcomes
[ ]	[x]	Estimates of effect sizes (e.g. Cohen's *d*, Pearson's *r*), indicating how they were calculated

Our web collection on statistics for biologists contains articles on many of the points above.

Software and code

Policy information about availability of computer code

Data collection	Raw ONT data was collected as multi-Fast5 or POD5 files using MinKNOW.
Data analysis	Rockfish code is publicly avaiable at: https://github.com/lbcb-sci/rockfish. Rockfish models are available at: https://zenodo.org/records/10867175. For basecalling we used Guppy basecaller (v5.0.14, sup mode) for R9.4.1 data and Dorado (v0.4.2) for R10.4.1 data. For the sequence alignment we used minimap2 (v2.24 for R9.4.1 data and v2.26 for R10.4.1 data). Rockfish R9.4.1 models were compared to Megalodon (v2.4.2) with Remora backend (v0.1.2) and Nanopolish (v0.14.0). Minimap2 (v2.24) was used both in Megalodon (via Mappy interface) and Nanopolish pipelines. Rockfish R10.4.1 model was compared to Remora (integrated in Dorado v0.4.2). For the WGBS methylation pipeline, we used Trim galore (v0.6.7) to perform quality and adapter trimming. After trimming, reads were processed using Bismark (v0.23.1). Running time evaluation was performed using GNU time command. See "Evaluation" subsection for more details.

For manuscripts utilizing custom algorithms or software that are central to the research but not yet described in published literature, software must be made available to editors and reviewers. We strongly encourage code deposition in a community repository (e.g. GitHub). See the Nature Portfolio guidelines for submitting code & software for further information.

Data

Policy information about availability of data

All manuscripts must include a data availability statement. This statement should provide the following information, where applicable:

- Accession codes, unique identifiers, or web links for publicly available datasets
- A description of any restrictions on data availability
- For clinical datasets or third party data, please ensure that the statement adheres to our policy

Both ONT and Illumina RRBS paired-end data for NA24385 is available via AWS at https://labs.epi2me.io/gm24385-5mc/. ONT NA12878 dataset is available via AWS at https://github.com/nanopore-wgs-consortium/NA12878. ONT data for NA12940 is available upon request from Chaisson et al. ref:Chaisson18. ONT data for HX1 is available at NCBI under project PRJNA533926 (https://www.ncbi.nlm.nih.gov/bioproject/?term=PRJNA533926). ONT data for K562 is available at Gene Expression Omnibus (GEO) under the BioProject GSE173688 (https://www.ncbi.nlm.nih.gov/geo/query/acc.cgi?acc=GSE173688).

WGBS paired-end data for NA12878 are available at ENCODE portal ref:sloan15 under accession numbers ENCFF798RSS (https://www.encodeproject.org/files/ENCFF798RSS/) and ENCFF113KRQ (https://www.encodeproject.org/files/ENCFF113KRQ/) (replicate 1), ENCFF585BXF (https://encodeproject.org/files/ENCFF585BXF/) and ENCFF851HAT (https://www.encodeproject.org/files/ENCFF851HAT/) (replicate 2). RRBS single-end data for NA12940 is available at ENCODE under accession numbers ENCFF000LZS (https://www.encodeproject.org/files/ENCFF000LZS/) (replicate 1) and ENCFF000LZT (https://www.encodeproject.org/files/ENCFF000LZT/) (replicate 2). WGBS paired-end data for HX1 is available at NCBI under the BioProject PRJNA301527 (https://www.ncbi.nlm.nih.gov/bioproject/PRJNA301527/). WGBS paired-end data for K562 is available at ENCODE under accession numbers ENCFF413KHN (https://www.encodeproject.org/files/ENCFF413KHN/) and ENCFF567DAI (https://www.encodeproject.org/files/ENCFF567DAI/) (replicate 1), ENCFF336KJH (https://www.encodeproject.org/files/ENCFF336KJH/) and ENCFF585HYM (https://www.encodeproject.org/files/ENCFF585HYM/) (replicate 2).

All newly sequenced data (ONT and WGBS for H1ESc, ONT for mouse datasets, WGBS for neonatal mouse dataset) are available at NCBI under BioProject PRJNA876781 (https://www.ncbi.nlm.nih.gov/bioproject/PRJNA876781/).

ChIP-seq data for NA12878 are available at ENCODE under accession numbers ENCSR000DZN (https://www.encodeproject.org/experiments/ENCSR000DZN/) (both two CTCF replicates and ChIP-seq control data). CHM13 excluded regions are available on GitHub (https://github.com/dozmorovlab/excluderanges).

Gene annotations (http://courtyard.gi.ucsc.edu/~mhauknes/T2T/t2t_Y/annotation_set_v2/CHM13.v2.0.cat_liftoff_v2.gff3) and RepeatMasker annotations (https://t2t.gi.ucsc.edu/chm13/hub/t2t-chm13-v2.0/rmsk/rmsk.bigBed) are downloaded from UCSC Genome Institute (UCSC GI). GC content data and CpG island annotations are downloaded from UCSC Baskin School of Engineering (https://hgdownload.soe.ucsc.edu/hubs/GCA/009/914/755/GCA_009914755.4/bbi/GCA_009914755.4_T2T-CHM13v2.0.gc5Base.bw).

Research involving human participants, their data, or biological material

Policy information about studies with human participants or human data. See also policy information about sex, gender (identity/presentation), and sexual orientation and race, ethnicity and racism.

Reporting on sex and gender	*Use the terms sex (biological attribute) and gender (shaped by social and cultural circumstances) carefully in order to avoid confusing both terms. Indicate if findings apply to only one sex or gender; describe whether sex and gender were considered in study design; whether sex and/or gender was determined based on self-reporting or assigned and methods used.*<br>*Provide in the source data disaggregated sex and gender data, where this information has been collected, and if consent has been obtained for sharing of individual-level data; provide overall numbers in this Reporting Summary. Please state if this information has not been collected.*<br>*Report sex- and gender-based analyses where performed, justify reasons for lack of sex- and gender-based analysis.*
Reporting on race, ethnicity, or other socially relevant groupings	*Please specify the socially constructed or socially relevant categorization variable(s) used in your manuscript and explain why they were used. Please note that such variables should not be used as proxies for other socially constructed/relevant variables (for example, race or ethnicity should not be used as a proxy for socioeconomic status).*<br>*Provide clear definitions of the relevant terms used, how they were provided (by the participants/respondents, the researchers, or third parties), and the method(s) used to classify people into the different categories (e.g. self-report, census or administrative data, social media data, etc.)*<br>*Please provide details about how you controlled for confounding variables in your analyses.*
Population characteristics	*Describe the covariate-relevant population characteristics of the human research participants (e.g. age, genotypic information, past and current diagnosis and treatment categories). If you filled out the behavioural & social sciences study design questions and have nothing to add here, write "See above."*
Recruitment	*Describe how participants were recruited. Outline any potential self-selection bias or other biases that may be present and how these are likely to impact results.*
Ethics oversight	*Identify the organization(s) that approved the study protocol.*

Note that full information on the approval of the study protocol must also be provided in the manuscript.

Field-specific reporting

[illegible]

Life sciences study design

All studies must disclose on these points even when the disclosure is negative.

Sample size	N.A.
Data exclusions	N.A.
Replication	N.A - no experimental work was done in this study. The results are obtained using the computational method.
Randomization	N.A. - no comparisons between multiple groups that would require randomization
Blinding	No blinding was performed in this study.

Reporting for specific materials, systems and methods

We require information from authors about some types of materials, experimental systems and methods used in many studies. Here, indicate whether each material, system or method listed is relevant to your study. If you are not sure if a list item applies to your research, read the appropriate section before selecting a response.

Materials & experimental systems

n/a	Involved in the study	
☒	☐	Antibodies
☐	☒	Eukaryotic cell lines
☒	☐	Palaeontology and archaeology
☐	☒	Animals and other organisms
☒	☐	Clinical data
☒	☐	Dual use research of concern
☒	☐	Plants

Methods

n/a	Involved in the study	
☒	☐	ChIP-seq
☒	☐	Flow cytometry
☒	☐	MRI-based neuroimaging

Eukaryotic cell lines

Policy information about cell lines and Sex and Gender in Research

Cell line source(s)	WiCell (hPSCReg ID: WAe001-A)
Authentication	Done by supplier
Mycoplasma contamination	Tested regularly and always negative
Commonly misidentified lines (See ICLAC register)	N.A.

Animals and other research organisms

Policy information about studies involving animals; ARRIVE guidelines recommended for reporting animal research, and Sex and Gender in Research

Laboratory animals	Species: Mus musculus; Strain: C57BL/6; Age: neonatal and 9 weeks for cardiomyocyte isolation, and 12 weeks for diet control mice blood collection
Wild animals	N.A.
Reporting on sex	For adult mice cardiomyocyte and diet control blood samples: only male mice were used in this study; For neonatal mice cardiomyocyte study: both genders were used
Field-collected samples	N.A.
Ethics oversight	All animal protocols and experiments were approved by the Institutional Animal Care and Use Committee at the National University of Singapore.

Note that full information on the approval of the study protocol must also be provided in the manuscript.

Plants

Seed stocks	*Report on the source of all seed stocks or other plant material used. If applicable, state the seed stock centre and catalogue number. If plant specimens were collected from the field, describe the collection location, date and sampling procedures.*
Novel plant genotypes	*Describe the methods by which all novel plant genotypes were produced. This includes those generated by transgenic approaches, gene editing, chemical/radiation-based mutagenesis and hybridization. For transgenic lines, describe the transformation method, the number of independent lines analyzed and the generation upon which experiments were performed. For gene-edited lines, describe the editor used, the endogenous sequence targeted for editing, the targeting guide RNA sequence (if applicable) and how the editor was applied.*
Authentication	*Describe any authentication procedures for each seed stock used or novel genotype generated. Describe any experiments used to assess the effect of a mutation and, where applicable, how potential secondary effects (e.g. second site T-DNA insertions, mosiacism, off-target gene editing) were examined.*
