# Supplementary material for: Rockfish: A transformer-based model for accurate 5-methylcytosine prediction from nanopore sequencing
Source: Nat Commun. 2024 Jul 3;15:5580. doi: 10.1038/s41467-024-49847-0 (PMC11222435; doi:10.1038/s41467-024-49847-0)
Supplement: Supplementary file 3 — Description of Additional Supplementary Files [file 41467_2024_49847_MOESM3_ESM.pdf]

Description of Additional Supplementary Files:

Supplementary Data 1: The table shows the results of the read-level evaluation for ONT-based tools on six different R9.4.1 datasets and two different R10.4.1 datasets. Only fully methylated or fully unmethylated sites w.r.t WGBS are included. Moreover, only examples that were predicted by all ONT tools are included in the evaluation. We evaluate five different contexts: (1) singletons and non-singletons, (2) genic regions (promoters, exons, introns, intergenic), (3) repetitive regions (LINE, SINE, LTR, DNA Transposons and other), (4) CpG islands, shores and shelves and (5) different GC contents (20%, 40%, 60%, 80%, 100%). For each evaluation, the number of evaluated examples is given in the column "Count". For each evaluation, we report five metrics: accuracy, precision, recall, false positive rate (FPR) and F1-score.

Supplementary Data 2: The table shows the results of the site-level evaluation for ONT tools on six different R9.4.1 datasets, and two different R10.4.1 datasets. Only fully methylated or fully unmethylated sites w.r.t WGBS are included. Moreover, only sites with coverage >= 5x for each ONT tool are included in the evaluation. We evaluate five different contexts: (1) singletons and non-singletons, (2) genic regions (promoters, exons, introns, intergenic), (3) repetitive regions (LINE, SINE, LTR, DNA Transposons and other), (4) CpG islands, shores and shelves and (5) different GC contents (20%, 40%, 60%, 80%, 100%). For each evaluation, the number of evaluated examples is given in the column "Count". For each evaluation, we report five metrics: accuracy, precision, recall, false positive rate (FPR) and F1-score. Empty cells denote NA (due to division with zero)

Supplementary Data 3: The table shows the results of the site-level correlation evaluation for ONT-based tools on six different R9.4.1 and two different R10.4.1 datasets. Sites with WGBS coverage >= 5x were included. Moreover, only sites with coverage >= 5x for each ONT tool are included in the evaluation. We evaluate five different contexts: (1) singletons and non-singletons, (2) genic regions (promoters, exons, introns, intergenic), (3) repetitive regions (LINE, SINE, LTR, DNA Transposons and other), (4) CpG islands, shores and shelves and (5) different GC contents (20%, 40%, 60%, 80%, 100%). For each evaluation, the number of evaluated examples is given in the column "Count". We use Pearson's r as a correlation measure.

Supplementary Data 4: The number of highly confident sites for WGBS and Rockfish small (RF). We define three types of highly confident positions (1) WGBS and Rockfish are concordant, (2) WGBS and Rockfish calls differ with the target tool having support from at least one other ONT-based method, (3) WGBS without support and Rockfish without call and vice-versa. A CpG site is defined as positive if the coverage is at least 5x and methylation frequency is at least 50%. A CpG site is defined as negative if the coverage is at least 5x and the frequency is less or equal to 50%. Sites with coverage less than 5x are labelled as not called. We performed this evaluation for the whole genome, genic regions and repetitive regions.

Supplementary Data 5: Execution time, CPU, GPU and memory usage for each ONT-base tool on the K562 R9.4.1 dataset. All commands were run three times. The range for each command is reported as (mean +/- std). The same basecalling evaluation (Command = Guppy) is reported for both Nanopolish and Rockfish. For Python-based tools (Megalodon Remora, Megalodon Rerio, Rockfish base, Rockfish small) computed peak memory usage represents a lower bound due to multiprocessing usage.

Supplementary Data 6: List of hyperparameters used for the Rockfish models. Rockfish small differs in the size of the features, the number of encoder and decoder layers and feed-forward dimension.

Supplementary Data 7: Distribution of proportions of positions not included in evaluation due low coverage (coverage threshold is set to 5). The positions with the insufficient coverage for Remora are split into two types: positions with overall coverage below threshold (counting modified, canonical and filtered call) - Remora proportion, and positions with valid coverage below threshold (counting only modified and canonical calls) but sufficient overall coverage - Remora filter proportion.

Supplementary Data 8: Distribution of proportions of high-filtering positions (HFP) with a sufficient valid coverage but above expected number of filtered calls (> 10%).

Supplementary Data 9: Site-level evaluation in CpG poor and CpG rich promoter regions for all positions and high-filtering positions (HFP).

Supplementary Data 10: Site-level evaluation in CpG poor and CpG rich promoter regions for all positions and high-filtering positions (HFP).

Supplementary Data 11: Execution time, CPU, GPU and memory usage for each ONT-base tool (R10.4.1) on the subset of NA12878 R10.4.1 dataset. All commands were run three times. The range for each command is reported as (mean +/- std). The same basecalling evaluation (Command = Dorado) is reported for both Rockfish with flash attention and Rockfish without flash attention. For Python-based tools (Rockfish) computed peak memory usage represents a lower bound due to multiprocessing usage.
